# Educational Case: Ectopic pregnancy and its relation to pelvic infections

**DOI:** 10.1016/j.acpath.2025.100168

**Published:** 2025-02-17

**Authors:** Liana Haven, Susan J. Roe

**Affiliations:** University of North Dakota School of Medicine and Health Sciences, Grand Forks, ND, USA

**Keywords:** Pathology competencies, Organ system pathology, Female reproductive, Disorders of pregnancy, Ectopic pregnancy, Pelvic infections, Pelvic inflammatory disease

The following fictional case is intended as a learning tool within the Pathology Competencies for Medical Education (PCME), a set of national standards for teaching pathology. These are divided into three basic competencies: Disease Mechanisms and Processes, Organ System Pathology, and Diagnostic Medicine and Therapeutic Pathology. For additional information, and a full list of learning objectives for all three competencies, see https://www.sciencedirect.com/journal/academic-pathology/about/pathology-competencies-for-medical-education-pcme.^1^

## Primary objective

Objective FDP1.1: Ectopic Pregnancy. Describe the risk factors, characteristic morphologic findings, potential outcomes, and the medical/surgical options for the management of ectopic pregnancy in relation to the pathogenesis and likelihood of adverse consequences.

Competency 2: Organ system pathology; Topic: Female reproductive—Disorders of pregnancy (FDP): Learning goal I: Disorders of pregnancy.

## Secondary objective

Objective FU4.1: Clinical features of pelvic infections. Discuss common pelvic infections, including those affecting the vulva, vagina, cervix, and fallopian tubes, and describe the pathogenesis of pelvic inflammatory disease, common organisms involved, and its complications.

Competency 2: Organ system pathology; Topic: Female reproductive—Uterus, cervix, and vagina (FU): Learning goal 4: Female genital tract.

## Patient presentation

A 23-year-old woman presents to the emergency department with right-sided lower abdominal pain. Her pain began insidiously last night and has increased drastically in the past 12 hours. The pain is only on the right side. She indicates that she has been mildly febrile, nauseated, and fatigued. She does not report having diarrhea, dark or bloody stools, dysuria, hematuria, or chills. She has a history of irregular menstrual cycles. Her last known menstrual period was 9 weeks ago. She began spotting two days prior thinking this was her cycle. However, she has only continued to spot and has not experienced her normal amount of menstruation. The pain is unlike previous menstrual cramps. She is sexually active and occasionally uses condoms for contraception. She is unsure if she has previously had a sexually transmitted infection (STI); she has not been tested in the past year.

## Diagnostic findings, Part 1

The patient’s temperature is 100.3° Fahrenheit. Pulse is 109 beats per minute and respiratory rate is 19 breaths per minute. Blood pressure is 104/62 mmHG. Oxygen saturation (SpO2) is 99 %. Her body mass index is 22.5 kg/m^2^. Physical exam shows regular heart rate and rhythm without murmurs and lungs clear to auscultation. There is extreme tenderness to palpation in the right lower quadrant of the abdomen with rebound tenderness, and no tenderness in the left quadrants of the abdomen. The abdomen is firm and without scars. Bowel sounds are present. McBurney’s sign is positive and Rosving sign is negative.

## Questions/discussion points, Part 1

### What is the differential diagnosis based on the history and physical examination?

Based on the location of the pain and the sudden onset, the main diagnoses that should be considered are acute appendicitis, ectopic pregnancy, or a ruptured ovarian cyst. Other diagnostic considerations include spontaneous abortion, ovarian torsion, a tubo-ovarian cyst or abscess, or acute pelvic inflammatory disease (PID). There would likely be no vaginal bleeding in the case of ovarian torsion, tubo-ovarian cyst or abscess, or appendicitis. Renal calculus or kidney stone can produce a similar sharp, sudden pain but frequently centers to flank pain rather than abdomen. Renal calculi may cause bleeding, although this blood would be seen in the urine and not from the vagina. Since the vaginal bleeding may be a light menses in a woman with known irregular periods, the differential should retain causes of lower abdominal pain without vaginal bleeding.

### What laboratory testing should be done?

A complete blood count (CBC), comprehensive metabolic panel (CMP), and urinalysis (UA) are ordered. The CBC is requested to see if there are indications of an infection or blood loss. The CMP is ordered to check liver and kidney function, electrolyte and fluid balance, and diabetes status. A urine pregnancy test is also requested.

## Diagnostic findings, Part 2

The urine pregnancy test is positive. CBC, CMP, and UA results are detailed in Table 1, Table 2, Table 3.

**Table 1:** Complete blood count.

		Reference range
White Blood Cell (WBC) Count	12 × 10^9^/L	4.5–11.0 × 10^9^/L
Red Blood Cell (RBC) Count	3.45 × 10^9^/L	3.5–5.5 × 10^9^/L
Hemoglobin (Hb)	11.0 g/dL	Female 12.0–16.0 g/dL
Hematocrit (HCT)	33.1 %	Female 36–46 %
Mean corpuscular volume (MCV)	82 fl	80-100 fl
Mean corpuscular Hgb (MCH)	29.2 pg/cell	25–35 pg/cell
Mean corpuscular Hb concentration (MCHC)	32.1 % Hb/cell	31–36 % Hb/cell
Red Cell distribution width (RDW)	12.6 %	12–15 %

**Table 2:** Comprehensive metabolic panel.

		Reference range
Sodium	139 mEq/L	136–145 mEq/L
Potassium	3.8 mEq/L	3.5–5.0 mEq/L
Chloride	100 mEq/L	95–105 mEq/L
Carbon dioxide	28 mEq/L	23–29 mEq/L
Anion gap	11 mEq/L	4–12 mEq/L
Urea nitrogen	13.5 mg/dL	7–18 mg/dL
Creatinine	0.71 mg/dL	0.6–1.2 mg/dL
Glomerular filtration rate estimate	>90	>60 (age variable)
Calcium	9.5 mg/dL	8.4–10.2 mg/dL
Glucose	91 mg/dL	70–110 mg/dL (fasting)

**Table 3:** Urinalysis.

		Reference range
Color	Light yellow	Yellow
Appearance	Clear	Clear or cloudy
Glucose	Negative	Negative
Bilirubin	Negative	Negative
Ketones	Negative	Negative
Specific gravity	1.02	1.002–1.035
Blood	Negative	Negative
pH	6.2	4.5–8
Protein albumin	Negative	</ = 150 mg/day
Urobilinogen	Normal	Small amount (0.5–1 mg/day)
Nitrite	Negative	Negative
Leukocyte esterase	Negative	Negative
Mucus	None seen	Small-moderate amount
RBC	1 cells/hpf	0-5 cells/hpf
WBC	0 cells/hpf	0-5 cells/hpf
Squamous epithelials	<1 cells/hpf	<15–20 cells/hpf

## Questions/discussion points, Part 2

### What is your interpretation of the findings in Table 1, Table 2, Table 3?

The CBC shows an elevated white blood cell count. Leukocytosis is a common finding with a broad differential. In this case, initial consideration is that it may be due to an infection or an inflammatory process. The hemoglobin and hematocrit are low and may indicate blood loss. The CMP is within normal limits. UA is within normal limits.

A serum hCG level is ordered in follow-up to the positive urine pregnancy test and found to be at 2,700 mIU/mL (an hCG level of <5 mIU/mL is considered negative for pregnancy and >25mIU/mL is considered positive. Retesting may be necessary in between those two levels).

***What imaging studies should be done?***

An ultrasound is requested as an initial imaging study to locate a possible pregnancy.

## Diagnostic findings, Part 3

**Fig. 1:**
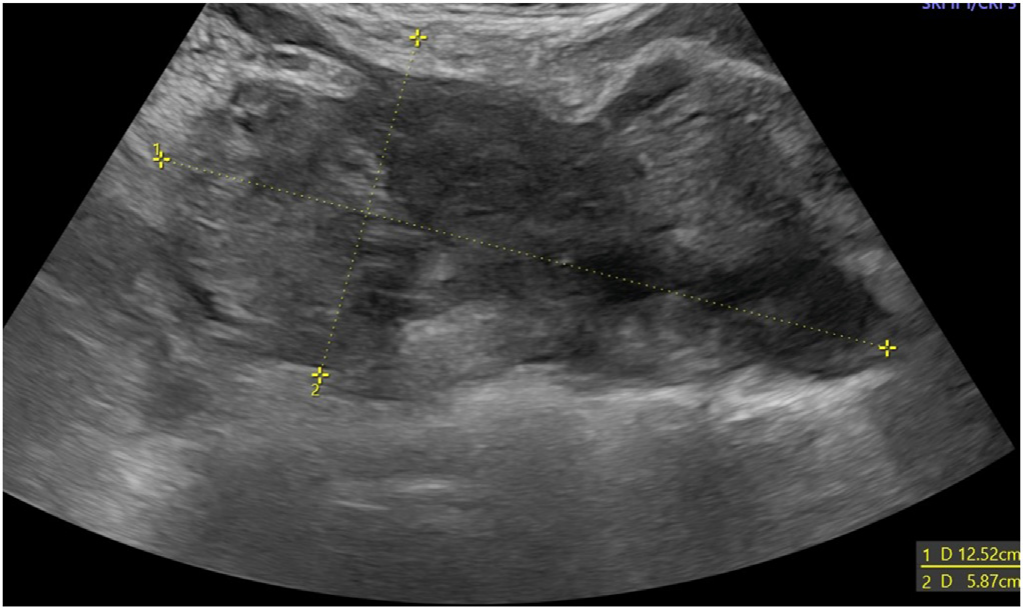
With transabdominal ultrasound, a large nonhomogeneous mass is seen in the right adnexa. Reprinted from content created by Dr. Salma Babikir Ahmed Erabi from Radiopaedia.org, rID:83742, under CC BY-NC-SA 3.0 license (https://creativecommons.org/licenses/by-nc-sa/3.0/).

**Fig. 2:**
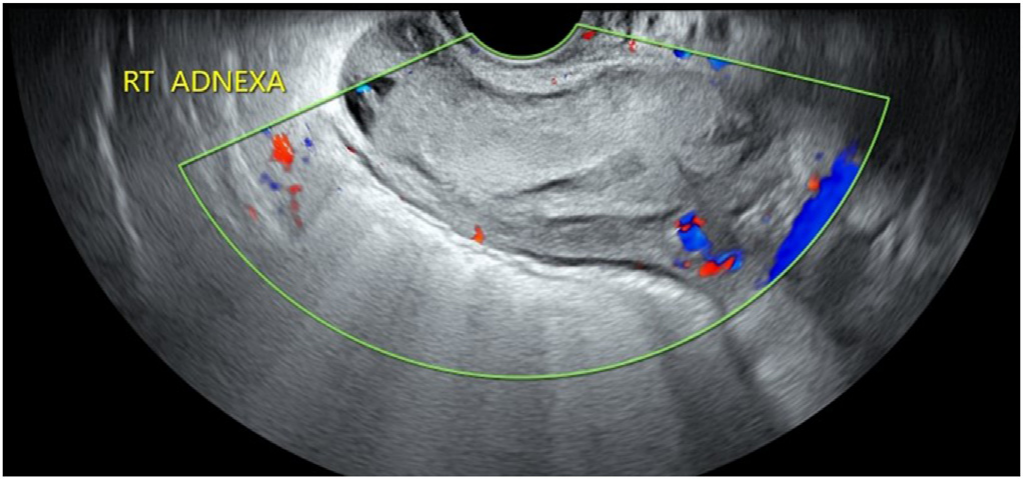
Transvaginal ultrasound using color Doppler demonstrates a nonhomogeneous mass in the right adnexa. Reprinted from content created by Dr. Salma Babikir Ahmed Erabi from Radiopaedia.org, rID:83742, under CC BY-NC-SA 3.0 license (https://creativecommons.org/licenses/by-nc-sa/3.0).

Ultrasound studies are shown in Fig. 1, Fig. 2.

## Questions/discussion points, Part 3

### What are the pertinent ultrasound findings?

There is a nonhomogeneous mass in the right adnexa. There is no intrauterine pregnancy.

### With the above laboratory findings and ultrasound studies, what is the most likely diagnosis?

A diagnosis of a ruptured ectopic pregnancy is made based on the right adnexal mass demonstrated on ultrasound, blood loss indicated by the low hemoglobin and hematocrit, and the physical exam findings of right lower quadrant pain with rebound tenderness. Preparation for surgery is initiated.

### How did additional testing contribute to making a specific diagnosis?

In this case, ultrasound is needed to locate the pregnancy and confirm if it is still active. Additionally, ultrasound is a quick and uncomplicated way to assess for fluid in the peritoneal space.^2^ Ultrasound, either transabdominal or transvaginal, is best for assessing reproductive organs/uterine structures. The CT is considered the standard of care when establishing a diagnosis of appendicitis.^3^ Ultrasonography in the pregnant patient has a poor specificity for ruling out appendicitis and there is a theoretical risk of ionizing radiation to the fetus. Therefore, magnetic resonance imaging (MRI), if available, is recommended rather than the CT in the pregnant patient. If MRI is not available and the diagnosis remains uncertain, a CT should be requested.^4^

### What are the most common risk factors for ectopic pregnancy?

Risk factors include a previous ectopic pregnancy, scarring of the tubal structures from infection, abdominal surgery, or endometriosis, and the presence of an intrauterine device (IUD). Although the likelihood of becoming pregnant with an IUD is extremely low, there is up to a 53 % chance of an ectopic pregnancy if one occurs. However, the risk of ectopic pregnancy with an IUD is less than that of using no contraception. Age over 35 years and tobacco use are lesser risk factors.^5^ An ectopic pregnancy may be seen in patients with no risk factors.

### What findings are expected on ultrasound and MRI that would be indicative of an ectopic pregnancy?

**Fig. 3:**
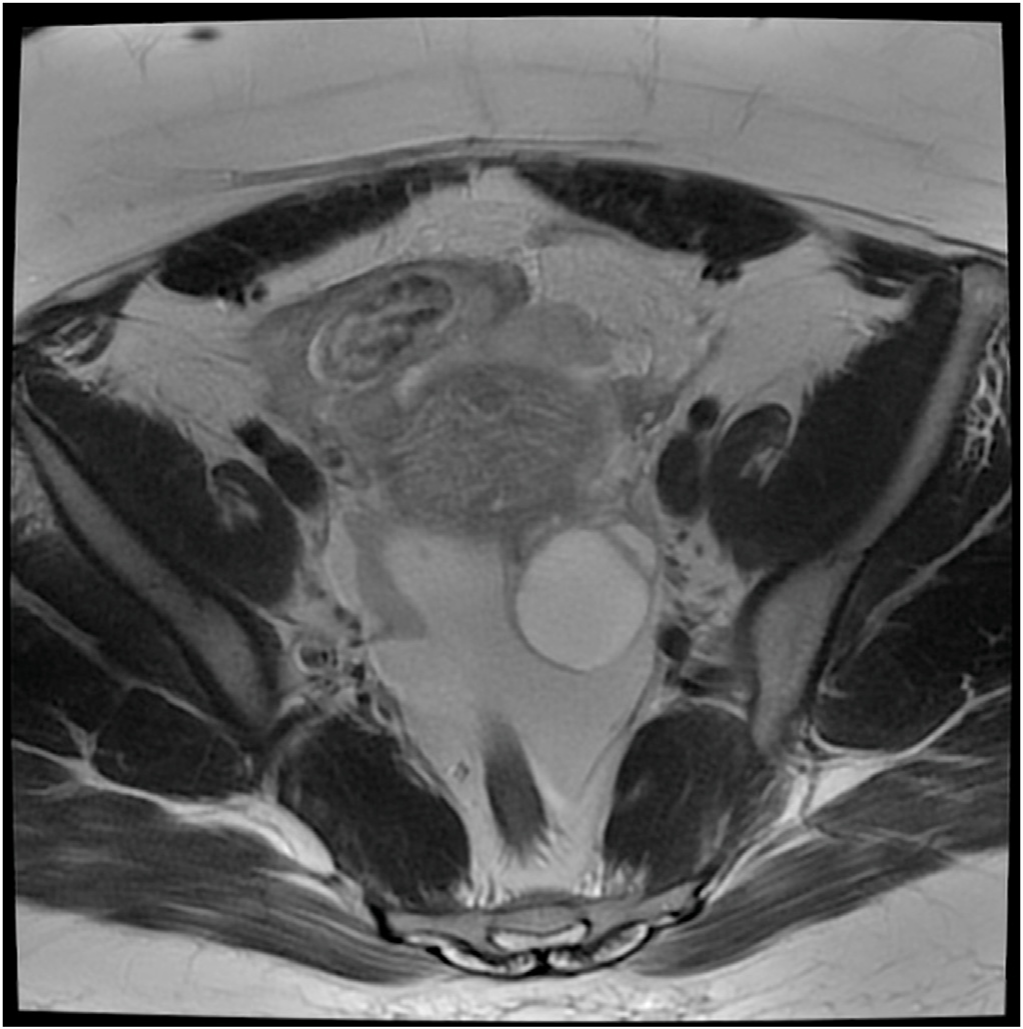
Axial T2 MRI demonstrates a right-sided tubal ectopic pregnancy with heterogenous internal signal. A low signal (white) cyst is noted from the left ovary. Reprinted from content created by Vikas Shah from Radiopaedia.org, rID:87383, under CC BY-NC-SA 3.0 license (https://creativecommons.org/licenses/by-nc-sa/3.0). MRI, magnetic resonance imaging.

**Fig. 4:**
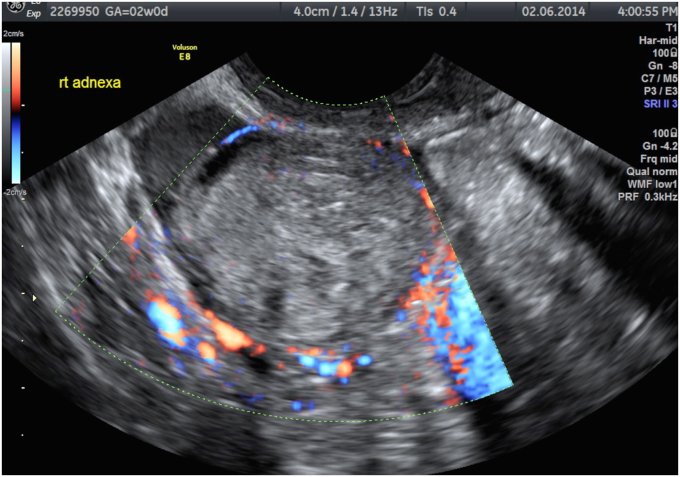
Ultrasound using color Doppler indicates a right adnexal mass with a “ring of fire” vascularity. Reprinted from content created by Alexandra Stanislavsky from Radiopaedia.org, rID:47667, under CC BT–NC–SA 3.0 license (https://creativecommons.org/licenses/by-nc-sa/3.0).

**Fig. 5:**
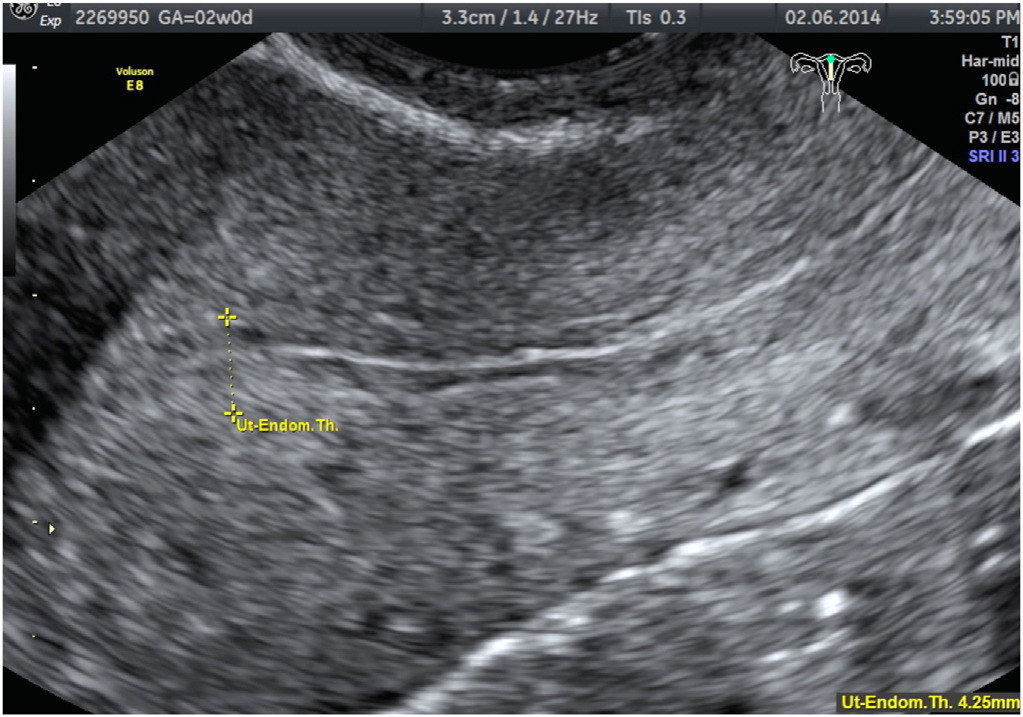
Ultrasound of uterus demonstrating empty uterine cavity and normal endometrial thickness. Reprinted from content created by Alexandra Stanislavsky from Radiopaedia.org, rID:47667, under CC BT-NC_SA 3.0 license (https://creativecommons.org/licenses/by-nc-sa/3.0).

With ectopic pregnancy, a common finding is the “ring of fire” sign (Fig. 3). This represents a ring of vascularity around an adnexal mass. Additionally, there should be an empty intrauterine space (Fig. 4), unless a heterotopic pregnancy is present.^2^ Ultrasound may also show if there is free fluid in the abdomen from rupture or bleeding from the pregnancy.^2^ There is often cardiac activity present as well. Doppler may show placental blood flow. However, this can be difficult to distinguish from surrounding structures of the corpus luteum.^2^ MRI may also define the ectopic tubal pregnancy (Fig. 5). If the ectopic pregnancy is secondary to PID, a CT correlation may show thickening of the uterosacral ligaments, pelvic fat stranding, reactive lymphadenopathy, or free fluid in the pelvis. However, some of these findings, such as the lymphadenopathy would only be present in the acute phase of PID.^6^

### Why is the patient prepared for emergency surgery?

A ruptured ectopic pregnancy can cause severe bleeding, infection, or death from the bleeding.

## Diagnostic findings, Part 4

**Fig. 6:**
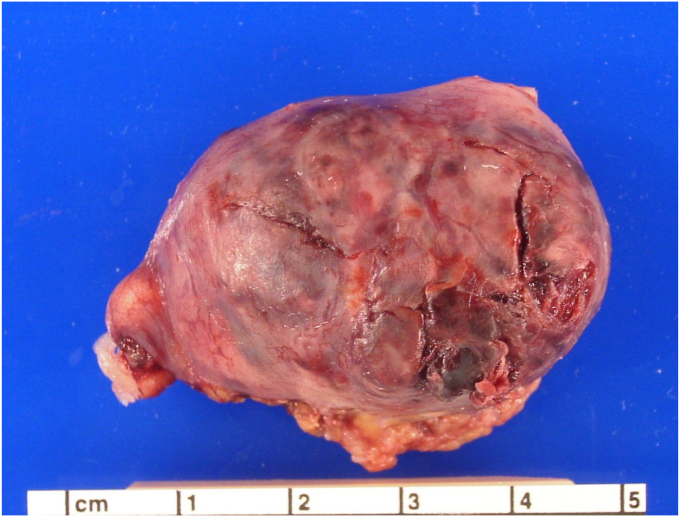
This salpingectomy specimen has a dilated fallopian tube with rupture and hemorrhagic tissue. (Used with permission of Mary Ann Sens, Ph.D., M.D., University of North Dakota School of Medicine and Health Sciences).

**Fig. 7:**
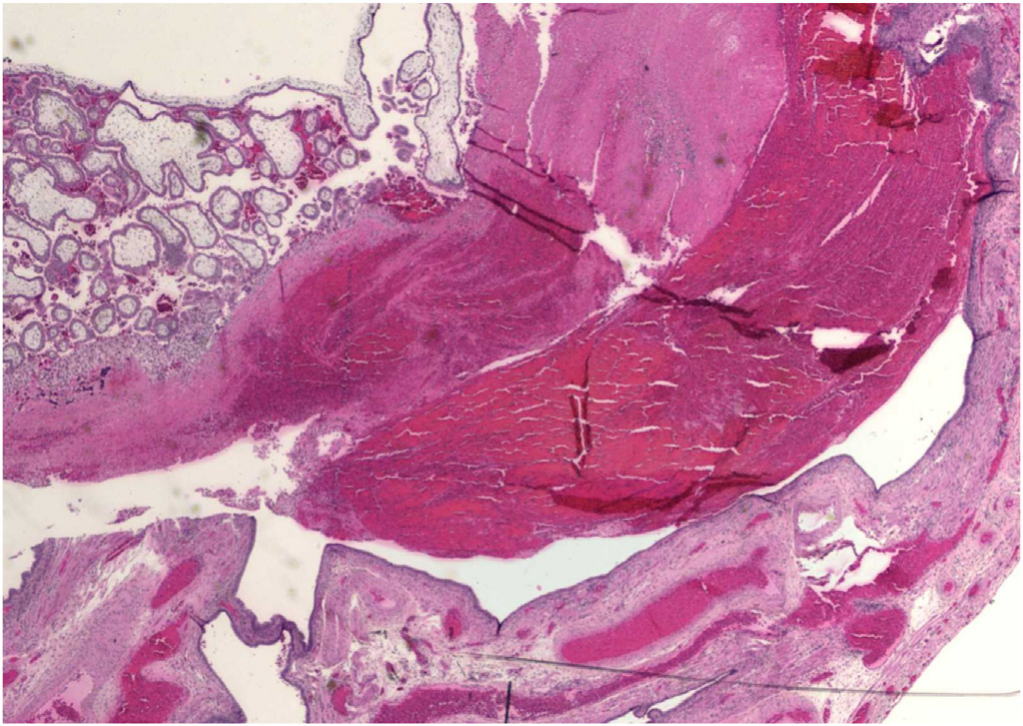
This is a low power H & E microscopic section of a ruptured ectopic pregnancy with blood clot and immature villi. No fetal parts are seen. (Used with permission of Mary Ann Sens, Ph.D., M.D., University of North Dakota School of Medicine and Health Sciences). H & E, **hematoxylin and eosin**

**Fig. 8:**
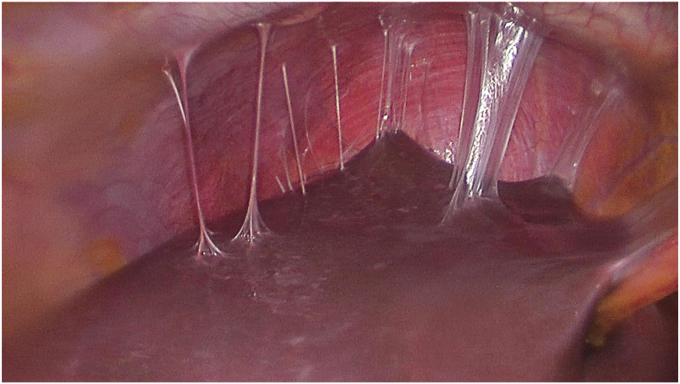
There are adhesions between the liver capsule and the diaphragm, representing Fitz–Hugh–Curtis syndrome (https://commons.wikimedia.org/wiki/File:Perihepatic_adhesions_2.jpg).

Due to the patient’s suspected ruptured ectopic pregnancy, she was taken to an operating room for emergent surgery. Laparoscopic salpingectomy was performed as the fallopian tube could not be salvaged because the pregnancy had already ruptured through the tube. The remainder of the right fallopian tube was removed to ensure no further damage could occur on that side. The peritoneal tissue was irrigated, adhesions lysed, and proper hemostasis was achieved. The surgical specimen of fallopian tube and ectopic pregnancy was sent to pathology for analysis (Fig. 6, Fig. 7). A view of the abdominal cavity showed adhesions of the liver capsule (Fig. 8).

## Questions/discussion points, Part 4

### What is your interpretation of the demonstrated pathology of the surgical specimen (Fig. 6, Fig. 7)?

The fallopian tube is markedly distended and there is a visible point of rupture on the surface. The histology shows blood and immature chorionic villi.

In the setting of a ruptured ectopic pregnancy, what do the liver capsule adhesions suggest to you?

The adhesions suggest Fitz–Hugh–Curtis Syndrome indicating a previous infection. The likely cause of the ectopic pregnancy is PID.

### What are symptoms, or physical exam findings, of acute PID?

If PID occurs acutely, there will likely be abrupt onset of lower abdominal pain either during or shortly after menses.^7^ There may be abnormal vaginal discharge or bleeding, dyspareunia, or dysuria. On physical exam, discharge may be noted coming from the cervix and the cervix may appear friable or inflamed indicating cervicitis. Cervical motion tenderness, or tenderness of any of the pelvic structures, can be a finding. It is important to note that many patients may be asymptomatic or have any combination of the symptoms above to varying degrees of severity. Some patients may also experience right upper quadrant pain caused by inflammation and adhesions (also referred to as Fitz–Hugh–Curtis Syndrome).^8^

***If this patient were presenting with symptoms related to PID, what other tests would be indicated?***

Nucleic acid amplification testing for both *Neisseria gonorrhoeae* (NG) and *Chlamydia trachomatis* (ChT) should be ordered from swab sampling done on pelvic exam. Testing for bacterial vaginosis could also be considered for another bacterial etiology that could ascend the genital tract.^8^ A pregnancy test should be ordered on all sexually active people with a uterus to exclude intrauterine or extrauterine pregnancy. Ultrasound or CT imaging could then be considered as well to better localize the pelvic pain and underlying cause.^7^

### How does PID increase the risk of ectopic pregnancy?

PID is an inflammatory response due to an infection. The most common pathogens to cause this reaction are NG and ChT. However, there are many other infectious etiologies such as those related to bacterial vaginosis or *Mycobacterium tuberculosis*.^8^ The process of PID begins with ascension of the pathogen from the lower genital tract into the upper genital tract through the cervix. This can lead to endometritis, salpingitis, or peritonitis. It may be asymptomatic, which is the case for many patients. Around 15 % of untreated chlamydial infections are estimated to progress to diagnosable PID with a higher risk of conversion to PID with gonococcal infection.^7^

The pathogens present within the upper genital tract will cause an influx of neutrophils to the area. However, due to pathogenic evasion, the neutrophils damage host cells instead of effectively removing the infected cells. This leads to cell damage or cell death, and loss of ciliation.^9^ Additionally, scar tissue can form as a result of the damage. When considering an ectopic pregnancy, one can see that lack of cilia would not propel the egg toward the uterus for proper implantation. The formation of scar tissue could further perturb the egg from implanting in the proper location.^7^

In the case of this patient, who had no recollection, or minimal symptoms from her prior infection, it could be stated that she had chronic PID (initial infection was greater than 30 days before noted pathologic findings).

### What treatment options would be recommended for an infectious cause of pelvic pain?

In the case of PID, broad spectrum antibiotics that cover both aerobes and anaerobes are recommended. The Pelvic Inflammatory Disease Evaluation and Clinical Health study found that the combination of cefoxitin and doxycycline provided good coverage for the variety of causes for PID and helped with mild-to-moderate PID. This treatment regimen cannot be used in pregnant individuals who would require hospitalization and IV antibiotics.^7^^,^^8^

### What other management options are there for ectopic pregnancy besides surgery?

Methotrexate is commonly used to treat ectopic pregnancies if caught early and the patient’s condition is stable. Methotrexate is a folate antagonist, which interrupts purine nucleotides and inhibits DNA synthesis and repair. This medication acts on rapidly dividing cells, like that of an embryo. Administration is typically intramuscular that requires close medical follow-up.^5^ Methotrexate cannot be prescribed to those with an intrauterine pregnancy, immunodeficiency, bone marrow dysfunction, elevated liver enzymes, or those with active gastrointestinal or respiratory diseases.^5^ There are three protocols that can be used for prescribing methotrexate for an ectopic pregnancy. They are the following:

1) Single dose: 50 mg/m^2^ IM on day 1 and measuring hCG on day 4 and day 7. Depending on hCG levels, a second dose may be required.
2) Two Dose: 50 mg/m^2^ IM on day 1 and on day 4. hCG levels checked on day 4 and day 7.
3) Fixed-multiple dose: 1 mg/kg IM on days 1, 3, 5, and 7 with alternating doses of IM folinic acid on days 2, 4, 6, and 8. The hCG is measured on each methotrexate dose day to verify a downward trend.

In the case of single and two dose regimens, hCG levels are expected to decrease by at least 15 % each time it is checked. In the case of the fixed-multiple dose regimen, methotrexate should be discontinued if there is a decrease in hCG levels by greater than 15 % as this means dosing is too high. Regardless of treatment protocol used, hCG levels must be trended down weekly until they are to nonpregnant levels. This is usually achieved by week 2–4 but can take up to 8 weeks.^5^ Close patient follow-up is required, which may be difficult for some patients to maintain. Although cost may be an issue, some patients may prefer surgery to avoid daily or weekly visits to the clinic. Another factor that patients should be counseled on is the need for efficacious birth control during this time and for 12 weeks after methotrexate use to avoid possible harm to a subsequent pregnancy.

Surgical options include salpingostomy with removal of the embryo from the tube, or complete removal of the fallopian tube (salpingectomy). For those undergoing salpingostomy, measuring hCG levels is needed and patients may still require a single dose of methotrexate to ensure all embryonic tissue is removed. Additionally, surgical options have a higher success rate, although they do carry the risk of any surgical procedure and use of anesthesia.^5^

Some ectopic pregnancies may end on their own in a spontaneous abortion. Serial hCG should be followed down with close monitoring to ensure rupture does not occur. Uterine aspiration may be done as well to remove any trophoblastic tissue present in the case of a failed intrauterine pregnancy, or pregnancy of unknown location if ultrasound cannot confirm location. If a fall in hCG is not seen after aspiration, this indicates either an ectopic location or incomplete evacuation of trophoblastic tissue.^5^

## Diagnostic findings, Part 5

The patient recovered well. Serial hCG performed post-op day 2 was down from 2,700 mIU/mL to 352 mIU/mL. She was discharged from the hospital with a birth control prescription. Serum hCG on post-op day 4 in outpatient clinic was undetectable. Her postoperative course was without complications. She healed well and reported feeling back to normal at her 6-week post-op visit. She continued to have yearly screening for ChT and NG as recommended by the Centers for Disease Control and Prevention. This would ensure that if reinfection were to occur, it would be detected early and treated effectively.

## Questions/discussion points, Part 5

### What are the current screening recommendations for sexually transmitted or pelvic infections for those with a uterus?

Currently, the CDC recommends all patients with a uterus who are 25 or younger and sexually active receive yearly screening for ChT and NG regardless of relationship status.^5^^,^^7^^,^^8^ Bacterial vaginosis does not fall into yearly screening and is only recommended to be tested for if a patient is symptomatic.^8^ For those over the age of 25, testing is recommended for those with new or multiple partners, inconsistent condom use, previous STI diagnosis, or if a partner had a positive STI test.^8^

## Teaching points

• There can be multiple causes for abdominal pain, including appendicitis, ectopic pregnancy, or a ruptured ovarian cyst. Evaluation of all potential causes is important for making a clear diagnosis.
• Imaging with ultrasound, MRI, or CT can help in having a clear diagnosis and rule out possible high-risk causes of abdominal pain. CT use in pregnancy is not a preferred option.
• On ultrasound, a nonhomogeneous adnexal mass, with or without cardiac activity, and an empty intrauterine space is highly indicative of an ectopic pregnancy.
• Characteristic morphologic findings of an ectopic pregnancy in a fallopian tube are a dilated, distended tube, possible rupture site, and the presence of blood clot, embryo, and chorionic villi in the tube.
• The outcomes of an ectopic pregnancy may include tubal rupture, which can lead to major internal bleeding. Some ectopic pregnancies may end on their own.
• Surgical options to treat an ectopic pregnancy include salpingostomy with removal of the products of conception or salpingectomy.
• Methotrexate is the nonsurgical treatment for ectopic pregnancy and requires extremely close follow-up, which may be a barrier to care for some patients.
• Patients should be followed and monitored closely for downward trending hCG levels and for signs of ruptured ectopic pregnancy, after methotrexate therapy.
• Risk factors for an ectopic pregnancy include a prior ectopic pregnancy, tubal scarring from infection, abdominal surgery or endometriosis, and the presence of an IUD.
• PID increases the risk for ectopic pregnancy through host-inflicted damage to cells. This can cause loss of ciliation and scar tissue formation within the fallopian tube preventing the embryo from implanting in the uterus.
• Treatment of PID includes coverage of aerobic and anaerobic pathogens.
• Yearly testing for *Neisseria gonorrhoeae* and *Chlamydia trachomatis* should be done by providers for sexually active patients, especially those under 25. Consistent condom use should also be discussed with patients even if they are on another form of birth control.

## Funding

The article processing fee for this article was funded by an Open Access Award given by the Society of ‘67, which supports the mission of the Association for Academic Pathology to produce the next generation of outstanding investigators and educational scholars in the field of pathology. This award helps to promote the publication of high-quality original scholarship in *Academic Pathology* by authors at an early stage of academic development.

## Declaration of competing interest

There are no conflicts of interest to declare on behalf of either Dr. Liana Haven or Dr. Susan Roe.
